# Soluble programmed cell death protein-1 and programmed cell death ligand-1 in sepsis

**DOI:** 10.1186/s13054-018-2064-3

**Published:** 2018-06-07

**Authors:** Debasree Banerjee, Sean Monaghan, Runping Zhao, Thomas Walsh, Amy Palmisciano, Gary S. Phillips, Steven Opal, Mitchell M. Levy

**Affiliations:** 1 Departments of Medicine, Warren Alpert Medical School of Brown University, Providence, RI USA; 2 Lifespan Hospital System, Providence, RI USA; 3 Department of Surgery, Warren Alpert Medical School of Brown University, Providence, RI USA; 4 Department of Biomedical Informatics, Center for Biostatistics, Ohio State University, Columbus, OH USA

**Keywords:** Sepsis, Soluble programmed cell death protein-1, Soluble programmed cell death ligand-1

Immunotherapy targeting the programmed cell death protein-1 (PD-1)–programmed cell death ligand-1 (PDL-1) axis in sepsis is poised for clinical trials, although optimal inclusion criteria and predictors of response are not well characterized.

We evaluated the kinetics of soluble (s)PD-1 and sPD-L1 in 30 septic intensive care unit (ICU) patients and 30 nonseptic ICU patients (Table 1). sPD-1 and sPD-L1 were significantly elevated among the septic cohort compared with the nonseptic ICU patients at enrollment (17.7 pg/ml vs. 4.5 pg/ml, *p* = 0.002; and 29.9 pg/ml vs. 11.3 pg/ml, *p* = 0.02; respectively) and were associated with sepsis (Fig. 1). Higher sPD-L1 on day 3 was associated with mortality among septic patients (16.7 pg/ml vs. 3.0 pg/ml, *p* = 0.054) and also in the total ICU cohort (14.9 pg/ml vs. 2.7 pg/ml, *p* = 0.026). Soluble PD-L1 regressed on interleukin (IL)-6 and interferon (IFN)γ levels were significantly associated in the total ICU cohort and septic patients, possibly pointing to upstream triggers for post-transcriptional modifications. Tumor necrosis factor (TNF)α regressed on sPD-1 and sPD-L1 was significant in all populations including septic survivors, revealing possible downstream effects of sPD-1 and sPD-L1. Initial sPD-1 levels correlated with a drop in lymphocyte count to < 1 × 10^9^/L (area under the receiver operating characteristic (ROC) curve 0.72, *p* = 0.006) and to < 0.6 × 10^9^/L (area under the ROC curve 0.68, *p* = 0.02). sPD-L1 also correlated with lymphocyte count drop to < 1 × 10^9^/L during the hospital stay. The correlation between the two immune checkpoint molecules, sPD-1 and sPD-L1, was also significant on enrollment, and at days 1 and 3 (*p* < 0.001, *p* < 0.001, *p* = 0.004, respectively; Fig. 2).

**Fig. 1:**
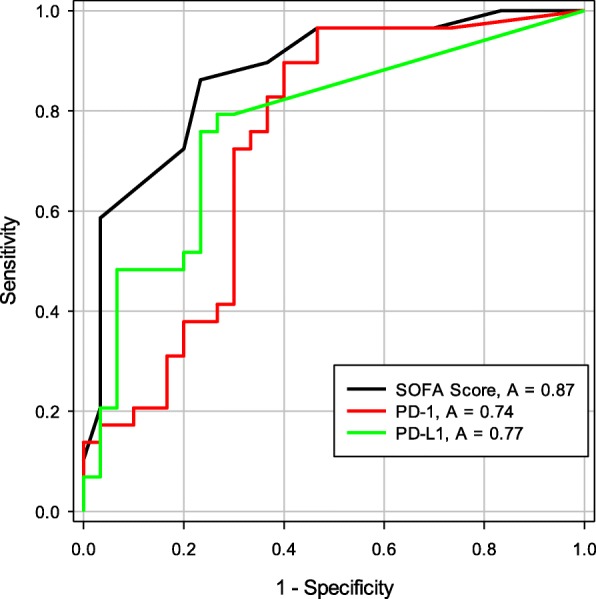
The area under the ROC curve for the discrimination of sepsis by soluble programmed death protein-1 (sPD-1) and soluble programmed death ligand-1 (sPD-L1). These are the day 0 area under the ROC curves for Sequential Organ Function Assessment (SOFA) score, sPD-1 (pg/ml), and sPD-L1 (pg/ml) for the discrimination of patients who have sepsis. Soluble PD-L1 outperforms sPD-1 for discrimination of sepsis

**Table 1:** Patient characteristics

Variables	Septic patients (*n* = 30)	Control subjects (*n* = 30)
Age (years), median (IQR)	63.3 (49.3–74.1)	58.6 (52.8–64.6)
Male, *n* (%)	11 (36)	19 (63)
White, *n* (%)	23 (76)	25 (83)
Past medical history		
COPD/asthma/fibrosis, *n* (%)	11 (36)	2 (7)
CAD/MI, CHF, AF, *n* (%)	16 (53)	7 (23)
Diabetes mellitus, *n* (%)	5 (17)	10 (33)
Malignancy, *n* (%)	8 (27)	6 (20)
CKD/ESRD, *n* (%)	5 (17)	1 (3)
Cirrhosis, *n* (%)	0 (0)	4 (13)
Connective tissue disease, *n* (%)	3 (10)	0
Arthritis, *n* (%)	3 (10)	3 (10)
Clinical assessment		
WBC (×10^−9^/L), median (IQR)	12.8 (6.9–19.1)	9.1 (7.6–10.9)
SOFA score, median (IQR)	7 (5–9)	2 (1–4)
Shock, *n* (%)	24 (80)	2 (6)
Site of infection in septic patients (*n*)		
Pneumonia	13	
Genitourinary tract infection	11	
Abdominal infection	2	
Meningitis	1	
Multiple sites of infection	1	
Bacteremia	9	
Unknown	1	
Organism of infection if known in septic patients (*n*)		
*Escherichia coli* not extended-spectrum β-lactamase producer	4	
*Escherichia coli* extended-spectrum β-lactamase producer	2	
Enterobacter	1	
*Enterococcus faecalis*	1	
*Acinetobacter baumannii*	1	
*Pseudomonas aeruginosa*	1	
Methicillin-resistant *Staphylococcus aureus*	1	
Methicillin-sensitive *Staphylococcus aureus*	1	
*Candida albicans*	1	
*Haemophilus influenzae*	1	
Bacillus species not anthracis	2	
*Klebsiella pneumoniae*	1	

*AF* atrial fibrillation, *COPD* chronic obstructive pulmonary disease, *CAD* coronary artery disease, *CHF* congestive heart Failure, *CKD* chronic kidney disease, *ESRD* end-stage renal disease, *IQR* interquartile range, *MI* myocardial infarction, *SOFA* sequential organ function assessment, *WBC* white blood cell

**Fig. 2:**
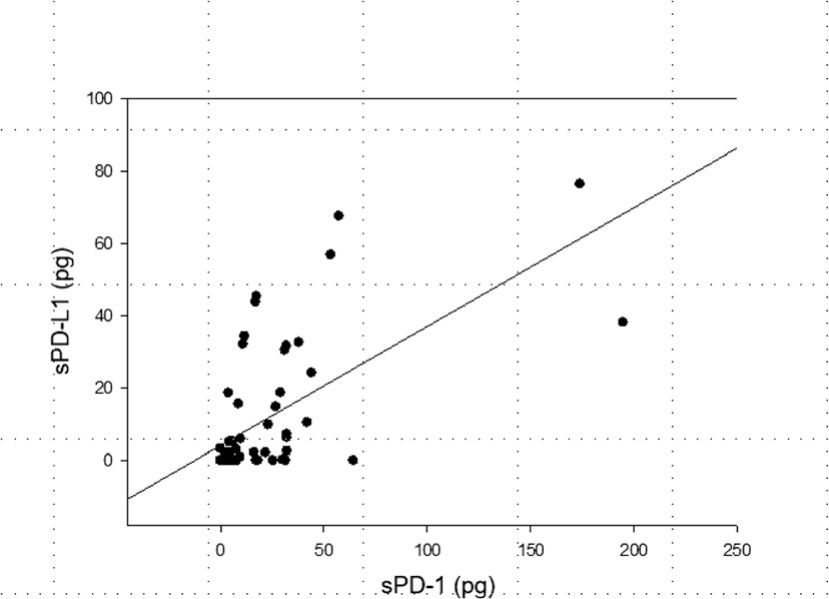
Correlation between soluble programmed death protein-1 (sPD-1) and soluble programmed death ligand-1 (sPD-L1) on day 0 among all ICU patients. The Pearson correlation between sPD-L1 (pg/ml on *y* axis) and sPD-1 (pg/ml on *x* axis) is 0.629 at the time of enrollment

sPD-1 and sPD-L1 are easily sampled, making them advantageous biomarkers in sepsis. A recent study demonstrated elevated sPD-1 among patients with infected pancreatitis [1]. sPD-1 may serve as an indicator of severity of sepsis among emergency room patients [2]. Lange et al. [3] reported that sPD-1 levels did not differ significantly between septic and nonseptic critically ill patients and had no association with outcome among septic patients. Our results may stem from sampling a different population to Lange and colleagues. Our controls, while critically ill, had lower severity of illness and mortality. Additionally, we excluded patients with immunocompromise, malignancy, and organ transplantation due to possible iatrogenic skewing of sPD-1 and sPD-L1. Approximately half of the control group in Lange et al. developed infections; thus, observations comparing sepsis versus nonseptic groups were limited to initial measurement only.

sPD-1 and sPD-L1 are point-of-care tests that might eventually guide personalized medicine in sepsis. These soluble immune checkpoints can risk-stratify patients for immunotherapy in sepsis and may potentially serve as targets themselves.

## Abbreviations

ICU: Intensive care unit
IFN: Interferon
IL: Interleukin
PD-1: Programmed cell death protein-1
PD-L1: Programmed cell death ligand-1
ROC: Receiver operator characteristic
TNF: Tumor necrosis factor
